# Healthcare outcomes and dispositions in persons with obesity within emergency departments in Ontario, Canada: A cross-sectional analysis of the National Ambulatory Care Reporting System (NACRS), 2018–2022

**DOI:** 10.1371/journal.pone.0311190

**Published:** 2024-09-26

**Authors:** Parmis Mirzadeh, Jennifer L. Kuk, Sean Wharton, Reagan A. Reid, Chris I. Ardern

**Affiliations:** 1 School of Kinesiology and Health Science, York University, Toronto, ON, Canada; 2 Wharton Medical Clinic, Toronto, ON, Canada; Universitair Kinderziekenhuis Koningin Fabiola: Hopital Universitaire des Enfants Reine Fabiola, BELGIUM

## Abstract

**Introduction:**

The experience of persons with obesity (PwO) in the Canadian healthcare setting has not been widely studied. The objective of this study was to assess care in PwO in emergency departments in Ontario, Canada.

**Methods:**

This secondary analysis made use of 2018–2022 Canadian Institute for Health Information’s National Ambulatory Care Reporting System. The sample consisted of 4547 individuals with an obesity diagnosis, and 4547 controls who were matched for sex, age, and main diagnosis. Ordinal logistic and multiple linear regression analyses were used to assess triage scores, wait times, and length of stay.

**Results:**

PwO had 4.8 minutes longer wait time for a physician initial assessment (p<0.01), 3.56 hours longer length of stay in the emergency department (p<0.0001), and 55% greater odds (OR = 1.55, 95% CI: 1.43–1.68) of having a less urgent triage score compared to controls matched for main diagnosis. When further matched for triage score, PwO experienced over three hours longer length of stay for triage level 2 (emergent, p<0.01), five hours longer for triage level 3 (urgent, p<0.01), and nearly two hours longer for triage level 4 (less urgent, p<0.05) cases.

**Conclusion:**

PwO were rated as less urgent and experienced longer wait times and length of stay, compared to controls matched by sex, age, and main diagnosis. Additional research is needed to confirm the consistency of these findings in other provinces/territories, and to examine clinical outcomes, and the underlying reasons for differences.

## Introduction

Obesity is a chronic, progressive, and relapsing disease which is characterized by excess body fat that can impair physical, mental, metabolic health as well as quality of life [1]. A multitude of health conditions are linked to obesity, many of which are frequently encountered within the emergency department setting in Canada.

Within the trauma setting, persons with obesity (PwO) are more likely to experience medical complications, organ failure, greater length of hospital stays, greater risk of death, and more days on mechanical ventilation [2–8]. These patterns have been demonstrated across different healthcare contexts, trauma severity, and research designs. In a prospective study of obesity and trauma outcomes in U.S. hospitals, PwO had more than a two-fold higher risk of admission to the Intensive Care Unit and risk of bloodstream, urinary tract, or respiratory infection, even after adjusting for age and Injury Severity Score [2]. In a retrospective cohort study of over 140 000 patients in the U.S., Glance et al. [4] found that PwO had a significantly higher risk of death and major complications, even after adjusting for injury severity and other risk factors. Finally, using a case-control design of patients admitted to a Level 1 trauma center in the U.S., Neville et al. [6] observed that although mechanisms and patterns of injury were similar, PwO had a higher incidence of multiple organ failure (13% vs 3%; p = 0.02) and mortality (32% vs 16%; p = 0.008). While these findings suggest worse overall outcomes in PwO, little is known about the underlying reasons and patterns of care in PwO.

Given differences between Canadian and U.S. health care models these U.S. findings require confirmation within the universal health care setting [9]. Moreover, the latest research on this topic [3,4] predates important changes to the treatment and management of obesity that have occurred since the recognition of obesity as a “disease” [10]. In addition to health system differences and potential temporal changes, Canadian and U.S. populations also differ in the sociodemographic patterning of obesity [11]. Furthermore, it is essential to assess patterns of care in the more recent healthcare context as PwO have been at a greater risk illness during the COVID-19 pandemic [12]. By using the Canadian Institute for Health Information’s 2018–2022 data, we will be able to examine these topics in greater detail within the Canadian healthcare context both prior to, and after, the onset of the COVID-19 pandemic. Given the high rate of obesity (and its comorbidity burden) in Canada, it is essential to examine differences in trauma-related outcomes to better understand needs for bariatric equipment and specialized care within Canadian hospitals. Thus, this study aims to explore patient care and health outcomes of PwO (vs matched controls) attending the emergency departments in the province of Ontario.

## Methods

### Study design and data source

This study is a secondary analysis of anonymized data accessed through the Graduate Student Data Access Program (GSDAP) of the Canadian Institute for Health Information (CIHI). CIHI is an independent not-for-profit organization that provides data architecture, analysis, and reports of routinely collected healthcare data in Canada. This study will use data derived from the CIHI National Ambulatory Care Reporting System (NACRS), representing serial presentations for all hospital emergency department visits. Ethics approval was obtained from the Human Participants Review Committee in the Office of Research Ethics at York University. Information regarding personal information privacy policy can be found on the CIHI website.

### Study sample and setting

The study sample was derived from CIHI NACRS Ontario emergency department visits occurring between April 1, 2018 and March 31, 2022, which includes administrative, clinical, and demographic information. NACRS captures data every fiscal year from April 1 to March 31; data for this study made use of pooled cycles from 2018–19, 2019–20, 2020–21, and 2021–22 (4547 case-control pairs, total n = 9094; age: 20 to 64 y). Duplicate records, any records relating to therapeutic abortions, cadaveric donors, and stillbirths were excluded. The cohort was selected using a case-control method, where cases have an obesity diagnosis (PwO, n = 4547 with ICD-10 E66) and controls were matched for age (within 5 years), sex, and main condition diagnosed in the emergency department within a given data cycle (n = 4547). In 6% of the control where the main condition of controls was unavailable, matching was done with age and sex only.

### Variables

Socio-demographic characteristics included sex (M, F), age (recategorized as: 20 to 44 and 45 to 64 y), income quintile, and area (urban or remote). Income was assessed by patient income quintiles based on census data of annual income [1 (lowest) to 5 (highest)]. A diagnosis of obesity was defined as one of five E66 codes: E66.0 Obesity due to excess calories, E66.1 Drug induced obesity, E66.2 Extreme obesity with alveolar hypoventilation, E66.8 Other obesity (morbid obesity), and; E66.9 Obesity, unspecified. Health service-related variables included triage levels captured by the Canadian Triage and Acuity Scale (CTAS; 1: severely ill, requires resuscitation, 2: requires emergent care and rapid medical intervention, 3: requires urgent care, 4: requires less-urgent care, 5: requires non-urgent care) which is a standard scale used in Canadian hospitals [13], wait time to physician initial assessment (hours), length of stay in the emergency department (hours), and responsibility for payment (provincial/territorial responsibility, other).

### Data analysis

Characteristics of the sample were compared between PwO and controls using conditional logistic regressions and paired t-tests. Multiple regression analyses were performed to assess differences in wait time to physician initial assessment and length of stay in the emergency department between PwO and controls. Ordinal logistic regression models were then used to estimate the odds of increasing triage level in PwO relative to controls (Unadjusted and Adjusted for sex, age, responsibility for payment, income, and area). Finally, mean wait time to physician initial assessment and length of stay in the emergency department were assessed between PwO and controls, matched by triage level (Canadian Triage and Acuity Scale, CTAS) using paired t-tests. To account for differences in emergency department use over the course of the pandemic, analyses were further stratified into pre- and post-onset of COVID-19 years (April 1, 2018 to March 31, 2020 vs. April 1, 2020 to March 31, 2022) using linear regression with time by PwO interactions terms.

## Results

**Table 1** shows the characteristics of PwO and matched controls. The majority of the sample was 45 to 64 years of age (65.4%), had lower income (Quintile 1, 31.9%), were from urban regions (85.1%), and had level 3 triage (require urgent care) emergency department visits (47.1%). Of the main conditions diagnosed, 21.6% of the sample had symptoms, signs and abnormal clinical and laboratory findings, 20.6% had diseases of the circulatory or respiratory system, and 10.4% had diseases of the digestive system.

**Table 1:** Characteristics of PwO and matched controls (n = 9094).

	Controls (n = 4547)	PwO (n = 4547)	p-value
**Sex**			
Female	2483 (54.61%)	2483 (54.61%)	
Male	2064 (45.39%)	2064 (45.39%)	
**Age**			
20 to 44 years old	1583 (34.81%)	1583 (34.81%)	
45 to 64 years old	2964 (65.40%)	2964 (65.40%)	
**Income quintile**			
1	1238 (27.23%)	1667 (36.66%)	
2	979 (21.53%)	958 (21.07%)	
3	889 (19.55%)	803 (17.66%)	
4	755 (16.60%)	664 (14.60%)	
5	686 (15.09%)	455 (10.01%)	p<0.001
**Area**			
Rural/Remote	692 (15.22%)	663 (14.58%)	
Urban	3855 (84.78%)	3884 (85.42%)	p = 0.39
**Triage level** *			
1	145 (3.19%)	182 (4.00%)	
2	1258 (27.67%)	1588 (34.92%)	
3	2124 (46.71%)	2160 (47.50%)	
4	781 (17.18%)	471 (10.36%)	
5	239 (5.26%)	146 (3.21%)	p<0.001
**Responsibility for payment**			
Provincial/territorial responsibility	4359 (95.87%)	4344 (95.54%)	
Other	188 (4.13%)	203 (4.46%)	P = 0.42

*Triage levels are based on the Canadian Triage and Acuity Scale (CTAS; 1: Severely ill, requires resuscitation, 2: Requires emergent care and rapid medical intervention, 3: Requires urgent care, 4: Requires less-urgent care, 5: Requires non-urgent care).

PwO: Persons with obesity.

p-values were obtained from conditional logistic regressions, there are no p-values for sex and age as controls and PwO were matched on these variables.

**Fig 1:**
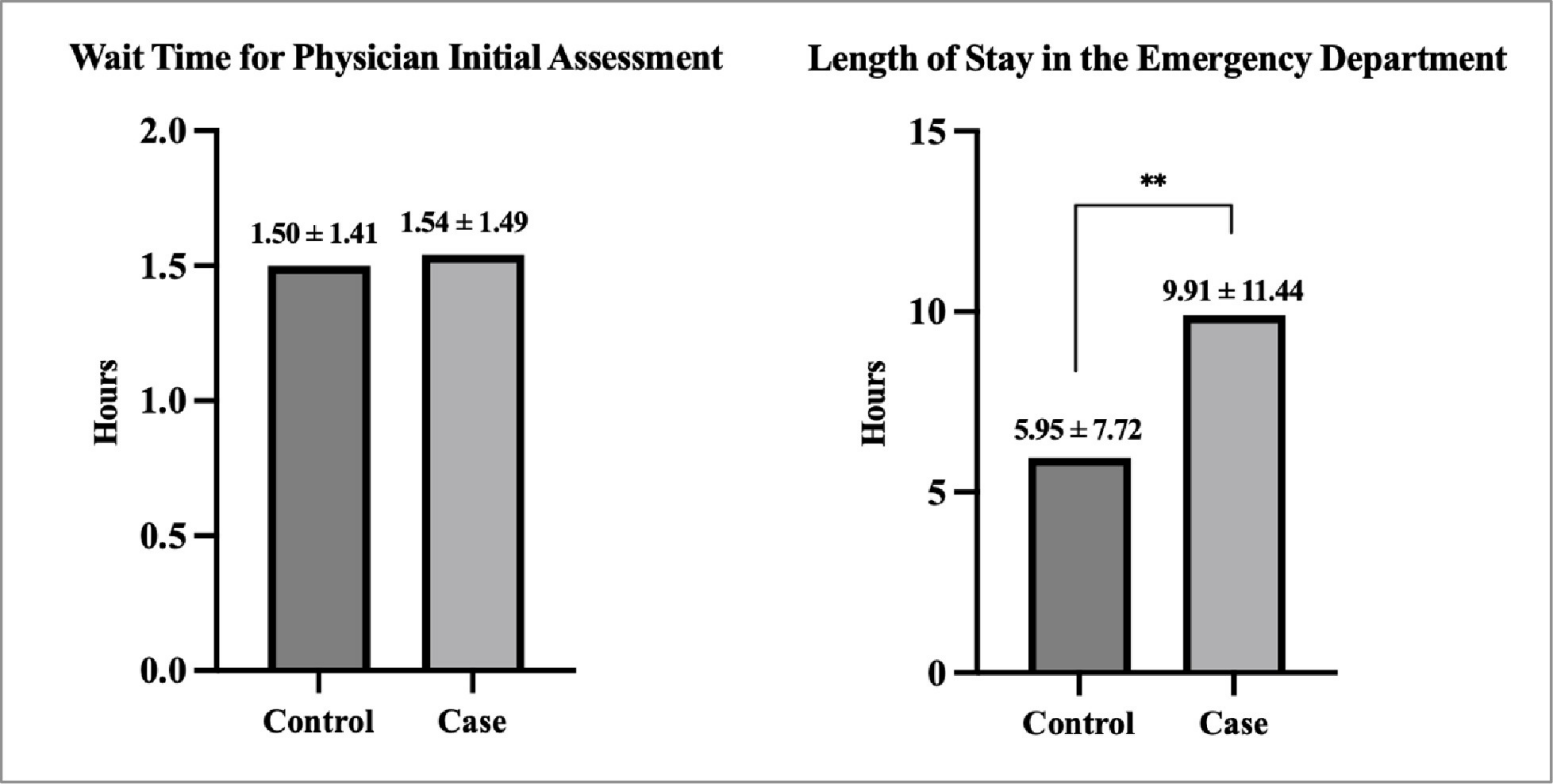
Mean wait time to a physician’s initial assessment and overall length of stay in the emergency department between PwO (case) and matched controls.

Paired t-tests revealed no difference in wait time to physician initial assessment (controls: 1.50±1.41 hours, cases: 1.54±1.49 hours, p = 0.19), but the average length of stay was significantly longer in PwO than controls (controls: 5.95±7.72 hours, cases: 9.91±11.4 hours, p<0.01, **Fig 1**). In models adjusted for sex, age, responsibility for payment, income, area, and triage level PwO had a 0.08 hour (4.8 minutes) longer wait time for a physician initial assessment (p<0.01), and 3.56 hours longer length of stay in the emergency department (p<0.0001), as compared to matched controls (**Table 2**).

**Table 2:** Results of regression analyses in wait time for physician initial assessment and length of stay in the emergency department in PwO relative to matched controls.

	Unadjusted		Adjusted	
	Beta ± SE	p-value	Beta ± SE	p-value
**Wait Time for Physician Initial Assessment** (hours, n = 8687)	0.04±0.03	0.16	0.08±0.03	<0.01
**Length of Stay in the Emergency Department** (hours, n = 9078)	3.96±0.20	<0.0001	3.56±0.20	<0.0001

Adjusted for sex, age, responsibility for payment, income, area, triage level.

Case: PwO, Control: matched for sex, age, main diagnosis.

**Fig 2:**
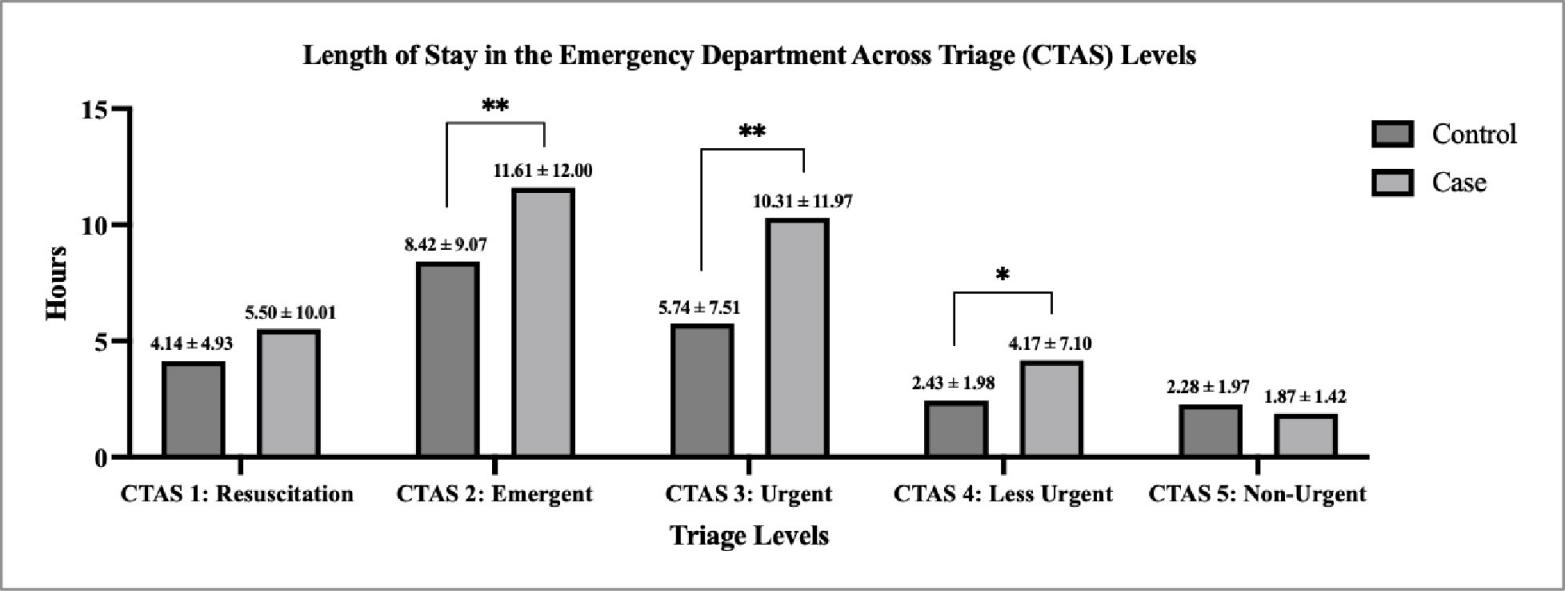
Mean length of stay in the emergency department between PwO (case) and controls matched across triage levels (Canadian Triage and Acuity Scale, CTAS).

**Table 3** displays results of the ordinal logistic regression analyses for triage levels across PwO and matched controls. In models adjusted for sex, age, responsibility for payment, income, and area PwO had a 55% greater odds of having a higher triage level (less urgent) compared to matched controls (OR = 1.55, 95% CI: 1.43–1.68). After further matching by triage level there were no significant differences for wait time to physician initial assessment (mean range, 0.1–15.0 minutes in triage level 1, p = 0.96, and triage level 5, p = 0.73) (**Fig 2)**. However, PwO had a length of stay in the emergency department that was more than three hours longer in triage level 2 (p<0.01), five hours longer for triage level 3 (p<0.01), and nearly two hours longer for triage level 4 (p<0.05) compared to matched controls.

**Table 3:** Odds of increasing triage levels in PwO relative to matched controls.

	Unadjusted OR (95% CI)	Adjusted OR (95% CI)
Control	1.00 (ref)	1.00 (ref)
Case (E66)	1.54* (1.43–1.67)	1.55* (1.43–1.68)

Adjusted for sex, age, responsibility for payment, income, area.

Case: PwO, Controls: Matched for age, sex, and main diagnosis.

Higher triage scores indicate less urgent cases.

OR = odds ratio (95% CI).

*Significance, p<0.05.

**Fig 3:**
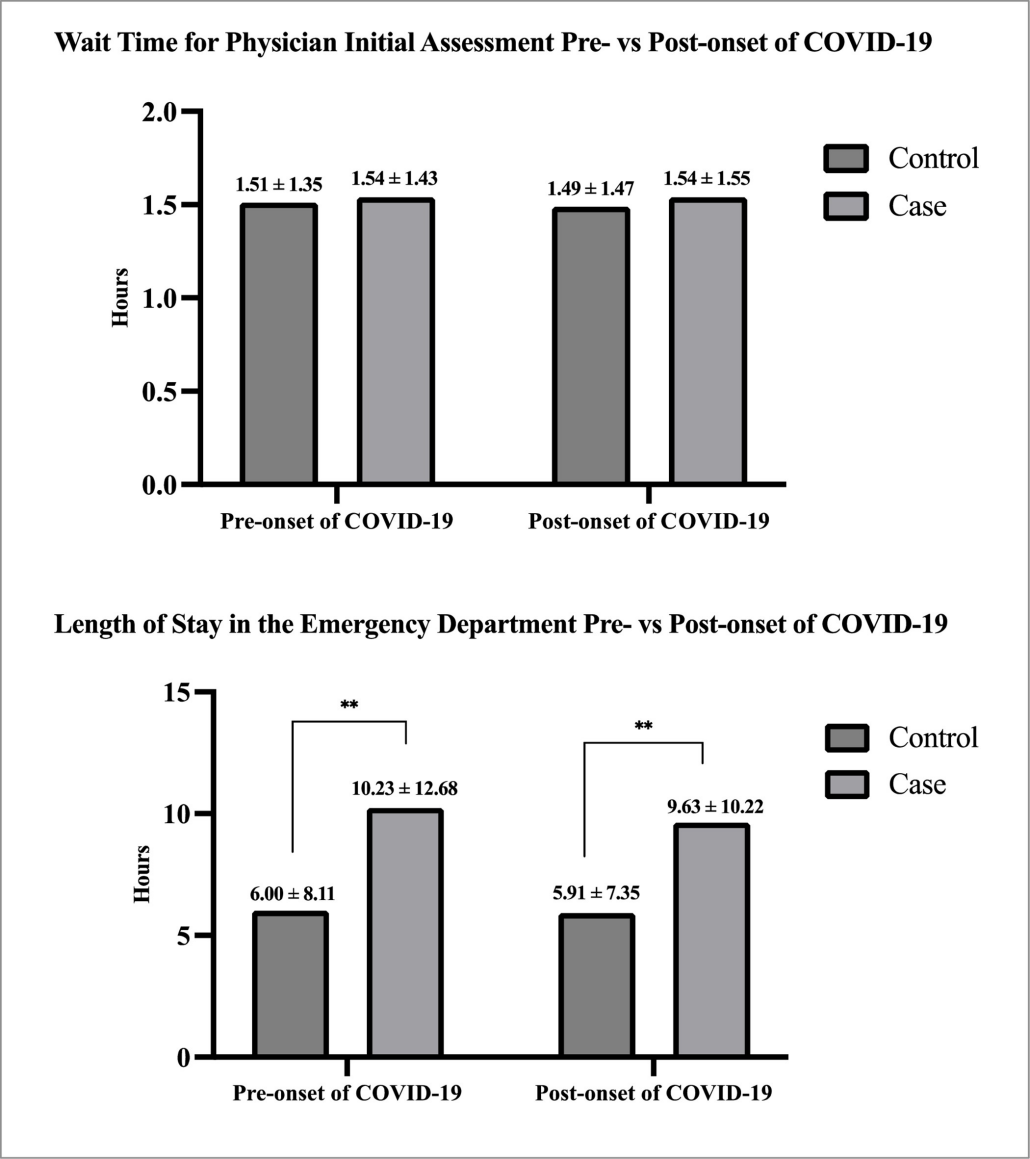
Mean wait time to a physician’s initial assessment and length of stay in the emergency department between PwO (case) and matched controls across time, examining pre- and post-onset of COVID-19 years.

**Fig 3** compares these relationships in the pre- and post-onset of COVID-19 years (April 1, 2018 to March 31, 2020 versus April 1, 2020 to March 31, 2022). In these analyses, wait time for physician initial assessment was not significantly different pre- versus post onset of COVID-19 for PwO and controls (p = 0.22). Furthermore no difference in wait time for physician initial assessment between PwO and controls in either the pre- (p = 0.67) or post-COVID 19 onset (p = 0.17) period; however, length of stay in the emergency department was significantly higher in PwO compared to matched controls, both pre- (controls: 6.00±8.11 hours, cases: 10.23±12.68 hours, p<0.01) and post-onset of COVID-19 (controls: 5.91±7.35 hours, cases: 9.63±10.22 hours, p<0.01), with shorter overall length of stay post-onset of COVID-19 (p<0.0001).

## Discussion

This study adds to existing literature on health and healthcare disparities for PwO by examining emergency department presentations from April 1, 2018, to March 31, 2022 in Ontario, Canada. Overall, this study found that PwO had a longer wait time to physician initial assessment, length of stay in the emergency department, and were rated with less urgent triage scores when compared to controls that were matched for sex, age, and main diagnosis. When further matched by triage level, the length of stay in the emergency department was greater in PwO across triage level 2 to 4 wherein PwO experienced on average two to five hours longer length of stay in the emergency department relative to controls. These findings have implications for bariatric healthcare preparedness and awareness in physicians.

In our current analysis, PwO were assigned higher triage scores, indicating lower priority issues than their matched controls, despite being matched for age, sex and main diagnosis. Triage scores are assigned based on patient complaint, type, and severity of signs and symptoms, to “ensure the sickest patients are seen first” [13]. Thus, PwO would be expected to have higher priority/more urgent triage scores given that they have obesity as an additional established comorbidity [14] which puts PwO at higher risk of serious diseases and health conditions [15–17]. The lower priority triage scores could be due in part to healthcare professionals attributing complaints, signs, and symptoms of PwO to obesity, rather than a potential underlying condition. For example, healthcare professionals might attribute a presentation of shortness of breath to an underlying respiratory condition in controls, but may attribute shortness of breath to obesity in PwO, leading to a lower priority score. Another possible explanation could be weight bias and stigma, where the negative ideologies around obesity contribute to discriminatory beliefs or actions towards PwO [18], which could result in the assignment of lower priority scores to PwO. Several reviews and meta-analyses have revealed weight bias and stigma to be evident across healthcare sectors and providers [19–21]. Key issues in healthcare utilization among PwO include lack of training, attribution of all health issues to excess weight, and avoidance or delay of health services from healthcare professionals [21]. Thus, it is possible that the negative ideologies surrounding obesity may have contributed to PwO being assigned a lower priority in the triage process.

In the present study, we observed that PwO experienced longer wait times for physician initial assessment and overall length of stay in the emergency department relative to controls matched for sex, age, and main diagnosis. These observations contrast with recent U.S. observational cohort study that found PwO had shorter wait times relative to patients with normal weight [22]. Potential explanations include inter-country differences in sociodemographic characteristics in private clinic data used in Lichen et al. (Mayo Clinic (U.S)) and our public provincial health care sample (Ontario, Canada), where wealthier individuals receiving care at the private U.S. clinic may have shorter wait times in general, or greater access to bariatric equipment, allowing for shorter wait times. The different operationalization of “obesity” in each study may also play a role: whereas Lichen et al. defined obesity by BMI, the present study defined PwO as those with an obesity diagnosis (E66), meaning the present study might include more severe cases of obesity, with greater health risk and higher BMI. However, if this were true, again, one would expect higher urgency scores and shorter wait times.

PwO had longer wait times and length of stay in adjusted analyses, and when matched by triage level, PwO still exhibited longer lengths of stay in the emergency department relative to controls in triage levels 2 to 4. Triage level 2 cases are considered “emergent”, with potential life-threatening conditions [13]; the fact that PwO had over three hours longer lengths of stay in the emergency department in triage level 2 could contribute to the overall worse outcomes in PwO within hospitals [2–8]. PwO also have a higher susceptibility to infections during their hospital stays [23,24], and longer wait times in emergency departments have been shown to be correlated with poorer clinical outcomes [25,26] and increased risk of mortality [27–29], highlighting the need to reduce exposure and risk of infection within this population. Given that timely treatment is an important predictor of health outcomes, these differences are concerning. The degree to which delays in treatment and longer lengths of stay are responsible for the differences in patient outcomes is unclear. Further research is necessary to understand whether PwO have longer wait times and stays because they require more care and treatment, or whether this is time spent waiting for care and treatment. It is also possible that these differences may be due to factors such as a greater difficulty in patient management in the emergency department for PwO, where higher BMI has been strongly correlated with difficulty finding anatomical landmarks, venous pressure measurement, physical examination, patient positioning, and procedures [30], thereby contributing to a delay in patient assessment and care. These findings highlight the need for a bariatric friendly healthcare system with specialized bariatric equipment [31] to better assess and care for PwO.

Finally, our finding that length of stay in the emergency department was overall lower across PwO and controls post-onset of the pandemic may be due to the decline in visits to Canadian Emergency Departments during the COVID-19 pandemic (36.9% decline, and 66% of expected volume) [32,33]. In particular, the longer length of stay in PwO relative to controls post-onset of COVID-19 may be due to PwO being found to be at higher risk of illness and poor prognosis due to COVID-19 [12], which may contribute to longer stays due to greater care and treatment required in PwO that present with COVID-19 symptoms.

Given that PwO experience prolonged wait times for physician initial assessment and longer stays in the emergency department, they should be a priority for interventions aimed at reducing emergency department wait times, and potentially reducing worse outcomes.

### Strengths and limitations

Taken together, this study provides new insight into the experiences of PwO within the emergency department setting in Ontario. Among the strengths of our analysis is our use of administrative health care data within a publicly funded universal healthcare system (i.e. “Medicare”) [34], which decreases risk of response bias differences across income groups. Indeed, the CIHI dataset used for the current analysis includes data from emergency department visits within Ontario hospitals (2018–19: n = 178; 2019–20: n = 178; 2020–21: n = 180, and; 2021–22: n = 179 facilities) over a four-year period. Second, PwO is defined as having an ICD-10 diagnosis of obesity (E66) by a physician during the emergency department encounter. By using E66 instead of BMI, the focus is on obesity-related health risk rather than weight alone, as BMI fails to account for variation in body composition and health risk by age, sex, and ethnicity [35]. Notwithstanding these study strengths, the study sample consists of emergency department visits in Ontario only, which may not generalize to the broader Canadian population or other jurisdictions. Even though obesity was defined using an E66 diagnosis code, it is possible that cases of obesity were missed due to a lack of diagnosis which would have biased results towards the null by their inclusion as a study control. It is also unclear how an E66 diagnosis was made, and whether the physician used measures such as BMI, waist circumference, comprehensive health or weight history, physical examinations, or other clinical tools in their assessment. Finally, controls were matched on sex, age, and main diagnosis only, as further matching was not statistically feasible. Instead, analyses were adjusted for possible confounders (i.e. responsibly for payment, income, and area).

## Conclusion

Even after matching for sex, age, and main diagnosis, this study demonstrates longer average wait times for physician initial assessment, length of stay in the emergency department, and triage scores indicating less urgent cases for PwO relative to controls. Future research is necessary to confirm the consistency of these findings in other Canadian provinces/territories and investigate clinical outcomes and the underlying reasons for disparities.

## References

[1] Obesity Canada. 2022. Available from: https://obesitycanada.ca.

[2] Bochicchio GV, Joshi M, Bochicchio K, Nehman S, Tracy KJ, Scalea TM. Impact of obesity in the critically ill trauma patient: A prospective study. Journal of the American College of Surgeons. 2006;203(4):533–8. Available from: https://www.sciencedirect.com/science/article/abs/pii/S1072751506010866?casa_token=8DTUSnaE41UAAAAA:EDDCYGSxyiKp92MbyChinzKJaGJH83aDU2vtMx4XNiJpKhE0GV 3fg_CCQWzuhmmcRO6wogaMiKI. doi: 10.1016/j.jamcollsurg.2006.07.001 17000398

[3] Ditillo M, Pandit V, Rhee P, Aziz H, Hadeed S, Bhattacharya B, et al. Morbid obesity predisposes trauma patients to worse outcomes: A National Trauma Data Bank analysis. J Trauma Acute Care Surg. 2014 Jan; 76(1):176–9. Available from: https://journals.lww.com/01586154-201401000-00026. doi: 10.1097/TA.0b013e3182ab0d7c 24368375

[4] Glance LG, Li Y, Osler TM, Mukamel DB, Dick AW. Impact of Obesity on Mortality and Complications in Trauma Patients. Ann Surg. 2014 Mar; 259(3):576–81. Available from: https://journals.lww.com/00000658-201403000-00024. doi: 10.1097/SLA.0000000000000330 24263314

[5] Ciesla DJ, Moore EE, Johnson JL, Burch JM, Cothren CC, Sauaia A. Obesity Increases Risk of Organ Failure after Severe Trauma. J Am Coll Surg. 2006 Oct; 203(4):539–45. Available from: https://journals.lww.com/journalacs/abstract/2006/10000/obesity_increases_risk_of_organ_failure_after.17.aspx. doi: 10.1016/j.jamcollsurg.2006.06.029 17000399

[6] Neville AL. Obesity Is an Independent Risk Factor of Mortality in Severely Injured Blunt Trauma Patients. Arch Surg. 2004 Sep 1; 139(9):983. Available from: http://archsurg.jamanetwork.com/article.aspx?doi=10.1001/archsurg.139.9.983. 15381617 10.1001/archsurg.139.9.983

[7] Brown CVR, Neville AL, Rhee P, Salim A, Velmahos GC, Demetriades D. The Impact of Obesity on the Outcomes of 1,153 Critically Injured Blunt Trauma Patients: J Trauma Inj Infect Crit Care. 2005 Nov; 1048–51. Available from: http://journals.lww.com/00005373-200511000-00003. 10.1097/01.ta.0000189047.65630.c5 16385276

[8] Byrnes MC, McDaniel MD, Moore MB, Helmer SD, Smith RS. The Effect of Obesity on Outcomes among Injured Patients: J Trauma Inj Infect Crit Care. 2005 Feb; 58(2):232–7. Available from: http://journals.lww.com/00005373-200502000-00002. 10.1097/01.ta.0000152081.67588.10 15706181

[9] Ridic G, Gleason S, Ridic O. Comparisons of Health Care Systems in the United States, Germany and Canada. Mater Sociomed. 2012; 24(2):112. Available from: https://www.ncbi.nlm.nih.gov/pmc/articles/PMC3633404/. doi: 10.5455/msm.2012.24.112-120 23678317 PMC3633404

[10] Wharton S, Lau DCW, Vallis M, Sharma AM, Biertho L, Campbell-Scherer D, et al. Obesity in adults: a clinical practice guideline. CMAJ. 2020;192(31):E875–91. Available from: doi: 10.1503/cmaj.191707 32753461 PMC7828878

[11] Adult obesity prevalence in Canada and the United States. Statistics Canada. 2015. Available from: https://www150.statcan.gc.ca/n1/pub/82-625-x/2011001/article/11411-eng.htm. 21592419

[12] CDC. Obesity, race/ethnicity, and COVID-19. Centers for Disease Control and Prevention. 2022. Available from: https://www.cdc.gov/obesity/data/obesity-and-covid-19.html.

[13] Prehospital Canadian Triage & Acuity Scale. Available from: https://www.lhsc.on.ca/media/2904/download.

[14] Lim Y, Boster J. Obesity and Comorbid Conditions. StatPearls Publishing; 2023. 34662049

[15] Guidelines (2013) for managing overweight and obesity in adults. Obesity (Silver Spring). 2014;22(S2):i–xvi. Available from: 10.1002/oby.20818. 24961823

[16] Clinical guidelines on the identification, evaluation, and treatment of overweight and obesity in adults—the evidence report. National institutes of health. Obes Res. 1998;6 Suppl 2:51S–209S. 9813653

[17] Bhaskaran K, Douglas I, Forbes H, dos-Santos-Silva I, Leon DA, Smeeth L. Body-mass index and risk of 22 specific cancers: a population-based cohort study of 5·24 million UK adults. Lancet. 2014;384(9945):755–65. Available from: 10.1016/s0140-6736(14)60892-8. 25129328 PMC4151483

[18] Weight stigma. World Obesity Federation. Available from: https://www.worldobesity.org/what-we-do/our-policy-priorities/weight-stigma.

[19] Lawrence BJ, Kerr D, Pollard CM, Theophilus M, Alexander E, Haywood D, et al. Weight bias among health care professionals: A systematic review and meta‐analysis. Obesity (Silver Spring). 2021;29(11):1802–12. Available from: doi: 10.1002/oby.23266 34490738

[20] Panza GA, Armstrong LE, Taylor BA, Puhl RM, Livingston J, Pescatello LS. Weight bias among exercise and nutrition professionals: a systematic review. Obes Rev. 2018;19(11):1492–503. Available from: doi: 10.1111/obr.12743 30176183

[21] Alberga AS, Edache IY, Forhan M, Russell-Mayhew S. Weight bias and health care utilization: a scoping review. Prim Health Care Res Dev. 2019;20(e116). Available from: doi: 10.1017/S1463423619000227 32800008 PMC6650789

[22] Lichen IM, Bellamkonda VR, Campbell RL, Phelan SM, Anderson JR, Mullan AF, et al. Association between patients’ body mass index and emergency department wait times: A multicenter observational cohort investigation by the reducing disparities increasing equity in emergency medicine (REDEEM) study group. Am J Emerg Med. 2021;49:178–84. Available from: doi: 10.1016/j.ajem.2021.06.007 34119812

[23] Falagas ME, Kompoti M. Obesity and infection. Lancet Infect Dis. 2006;6(7):438–46. Available from: doi: 10.1016/S1473-3099(06)70523-0 16790384

[24] Dossett LA, Dageforde LA, Swenson BR, Metzger R, Bonatti H, Sawyer RG, et al. Obesity and site-specific nosocomial infection risk in the intensive care unit. Surg Infect (Larchmt). 2009;10(2):137–42. Available from: doi: 10.1089/sur.2008.028 19388836 PMC2963594

[25] Byrne D, Browne JG, Conway R, Cournane S, O’Riordan D, Silke B. Mortality outcomes and emergency department wait times—the paradox in the capacity limited sytem. Acute Med. 2018;17(3):130–6. 30129945

[26] Paton A, Mitra B, Considine J. Longer time to transfer from the emergency department after bed request is associated with worse outcomes. Emerg Med Australas. 2019;31(2):211–5. Available from: doi: 10.1111/1742-6723.13120 30129706

[27] Jones S, Moulton C, Swift S, Molyneux P, Black S, Mason N, et al. Association between delays to patient admission from the emergency department and all-cause 30-day mortality. Emerg Med J. 2022;39(3):168–73. Available from: doi: 10.1136/emermed-2021-211572 35042695

[28] Plunkett PK, Byrne DG, Breslin T, Bennett K, Silke B. Increasing wait times predict increasing mortality for emergency medical admissions. Eur J Emerg Med. 2011;18(4):192–6. Available from: doi: 10.1097/MEJ.0b013e328344917e 21317786

[29] Singer AJ, Thode HC Jr, Viccellio P, Pines JM. The association between length of emergency department boarding and mortality: Boarding and mortality. Acad Emerg Med. 2011;18(12):1324–9. Available from: 10.1111/j.1553-2712.2011.01236.x. 22168198

[30] Kam J, Taylor DM. Obesity significantly increases the difficulty of patient management in the emergency department: Effects of obesity. Emerg Med Australas. 2010;22(4):316–23. Available from: 10.1111/j.1742-6723.2010.01307.x. 20796008

[31] Pearce N. Bariatric friendly health care service [Internet]. Obesity Canada. Available from: https://obesitycanada.ca/resources/bariatric-friendly/.

[32] Kwok ESH, Clapham G, Calder-Sprackman S. The impact of COVID-19 pandemic on emergency department visits at a Canadian academic tertiary care center. West J Emerg Med. 2021;22(4):851–9. Available from: doi: 10.5811/westjem.2021.2.49626 35353999 PMC8328159

[33] Lee DD, Jung H, Lou W, Rauchwerger D, Chartier LB, Masood S, et al. The impact of COVID-19 on a large, Canadian community emergency department. West J Emerg Med. 2021;22(3):572–9. Available from: doi: 10.5811/westjem.2021.1.50123 34125029 PMC8202991

[34] Canada.ca. Available from: https://www.canada.ca/en/health-canada/services/canada-health-care-system.html.

[35] Bmi W is. Body mass index: Considerations for practitioners. Cdc.gov. Available from: https://stacks.cdc.gov/view/cdc/25368.

